# Supplementary material for: Fibre optic distributed acoustic sensing of volcanic events
Source: Nat Commun. 2022 Mar 31;13:1753. doi: 10.1038/s41467-022-29184-w (PMC8971480; doi:10.1038/s41467-022-29184-w)
Supplement: Supplementary file 1 — Supplementary Information [file 41467_2022_29184_MOESM1_ESM.pdf]

Fibre optic distributed acoustic sensing of volcanic events

P. Jousset et al.,

Supplementary Information

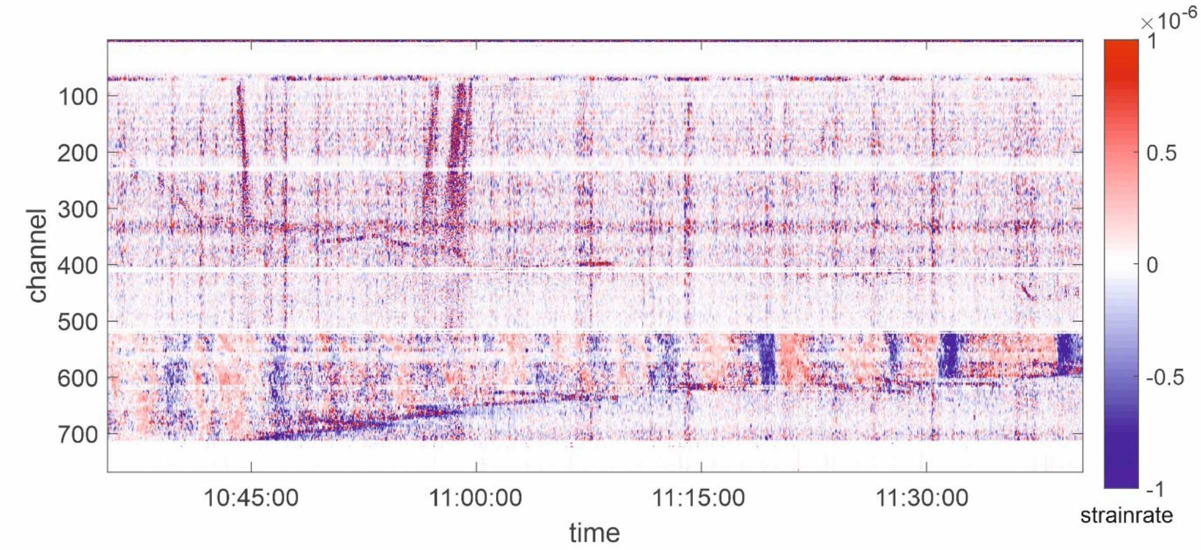

Supplementary Fig. 1 | Cable deployment. The cable is deployed both at depth (15-25 cm) and at the surface (see Fig. 1 for details of the channel correspondence and Supplementary Note 2: Coupling between cable and scoriae). The time section (about 1 hour) shows distributed acoustic sensing (DAS) records in the morning of September 1, 2018, when we covered the superficial cable with several centimetres of scoriae, starting near the end of the cable (channel 700) at 10:43 and progressively making our way to smaller channel numbers. As the shallow cable is covered, the amplitude of the low frequency noise (possibly corresponding to wind) is significantly reduced and signals on both the deep and the shallow cables are then comparable, e.g., during transients. However, for some frequencies and depending on the source process, we noticed smaller amplitudes in the records of the deep cable than those closer to the surface.

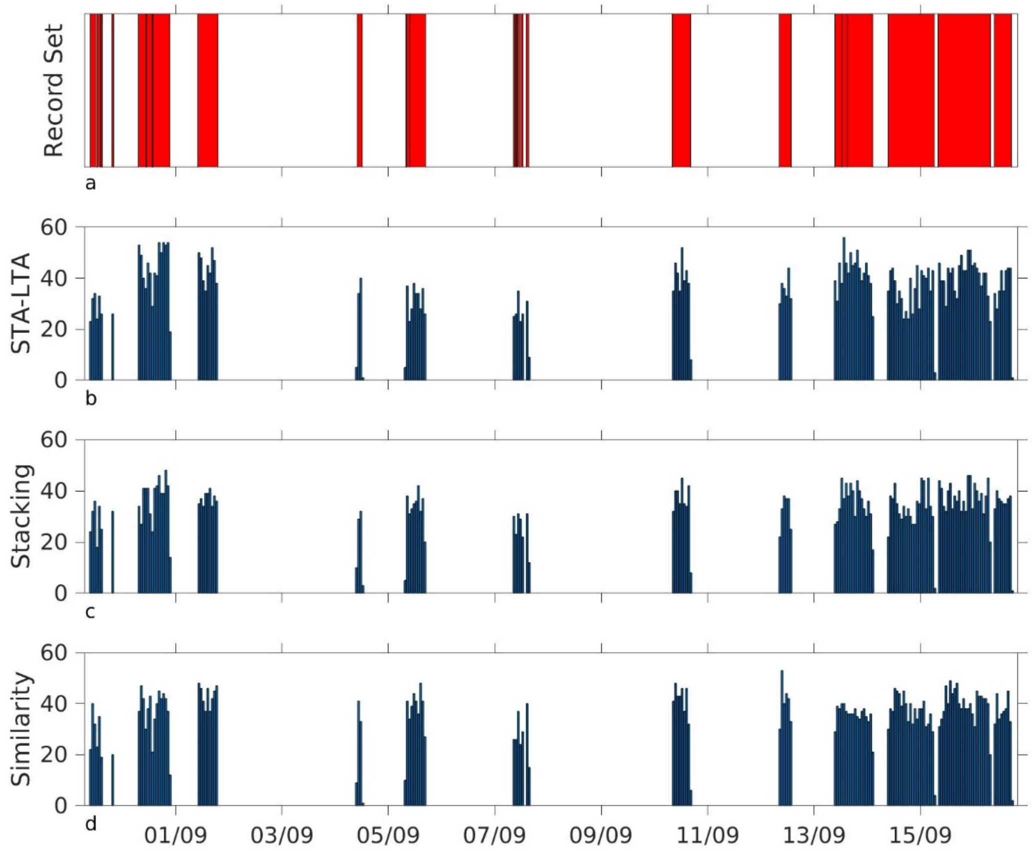

Supplementary Fig. 2 | Record set and event detections during the experiment. a. Record set (red) as a function of time. Hourly event detection for three detection methods **b.** Short-term Average (STA) - Long-term average (LTA) detection; **c.** Amplitude stacking; **d.** Local similarity. The detection methods are detailed in Method: detection methods for DAS continuous monitoring.

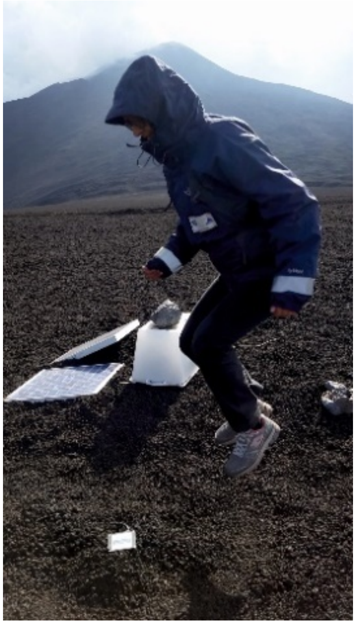

a.

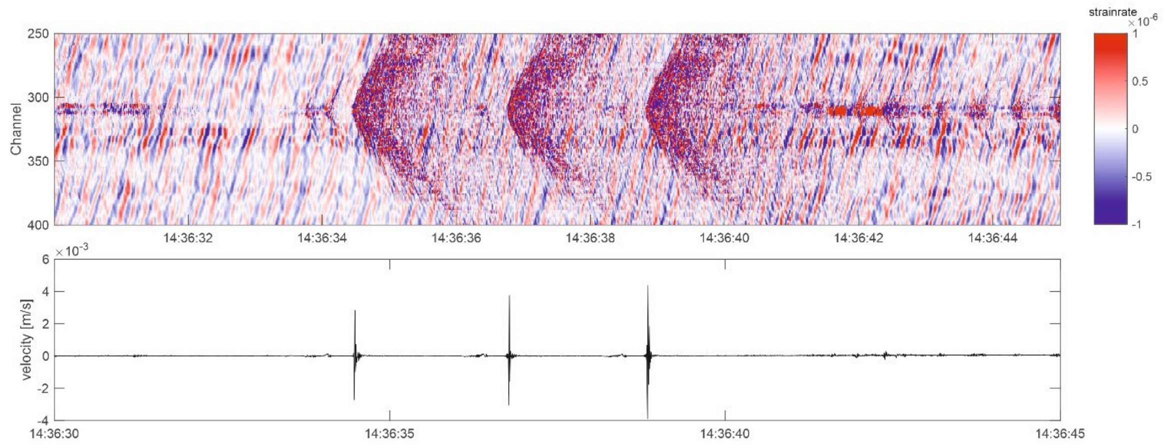

b.

Supplementary Fig. 3 | Distributed acoustic sensing (DAS) records of jumps along the cable. a. Example of a jump performed along the cable near a broad-band sensor (white box with solar panels), and the geophone C272 (with a cube). **b. Top.** Strain rate records for channels 250-400. The surface waves produced by the jump propagate along the cable over many traces (up to 150 meters distance). In the background, volcanic tremor is visible as parallel stripes with amplitude +/- 0.5×10^{-6} s^{-1} (strain rate). **Bottom.** Ground velocity recorded by the closest geophone C272.

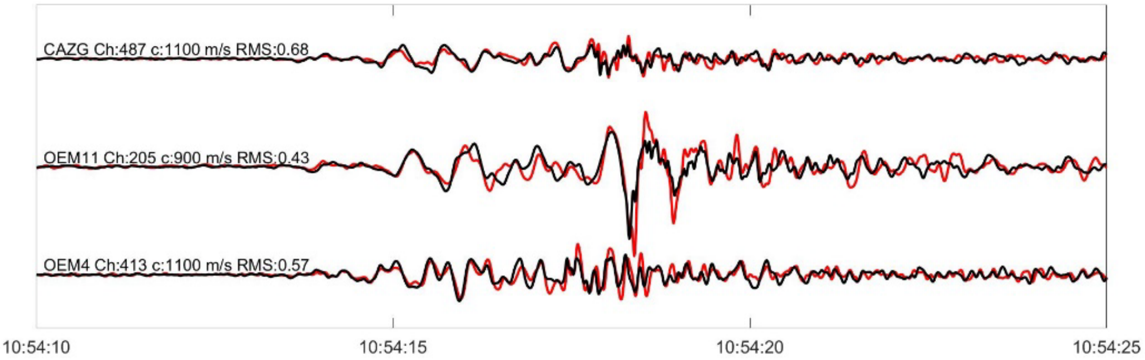

a.

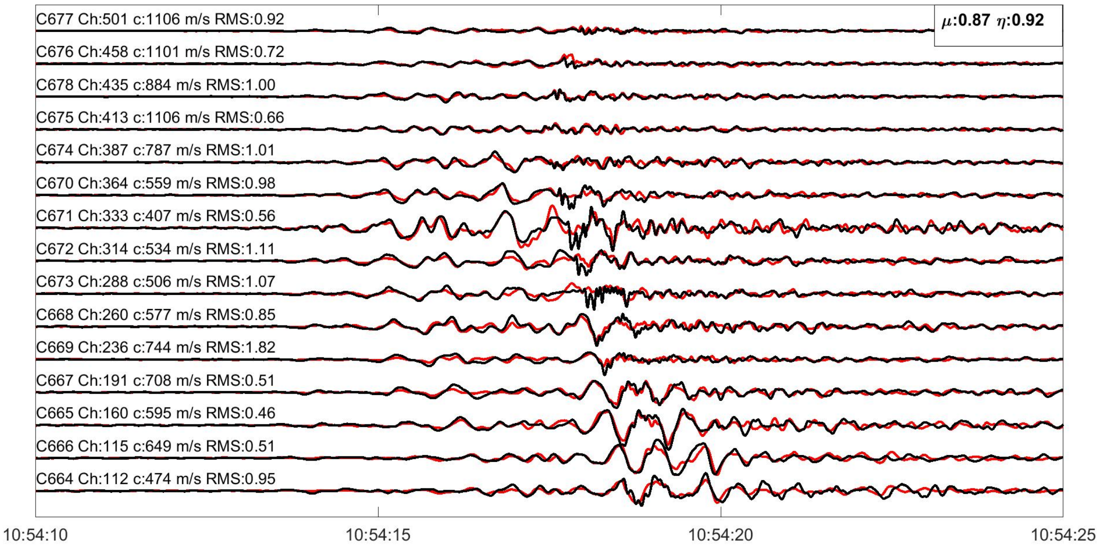

b.

Supplementary Fig. 4 | DAS strain validation. Strain estimates from seismic data (black) compared to strain recorded by DAS on the deep cable (red). Strain estimated **a.** for individual broadband seismometers[1] (Method: DAS strain rate and strain validation, phase velocity), **b.** for individual geophones (Method: DAS strain rate and strain validation, phase velocity)

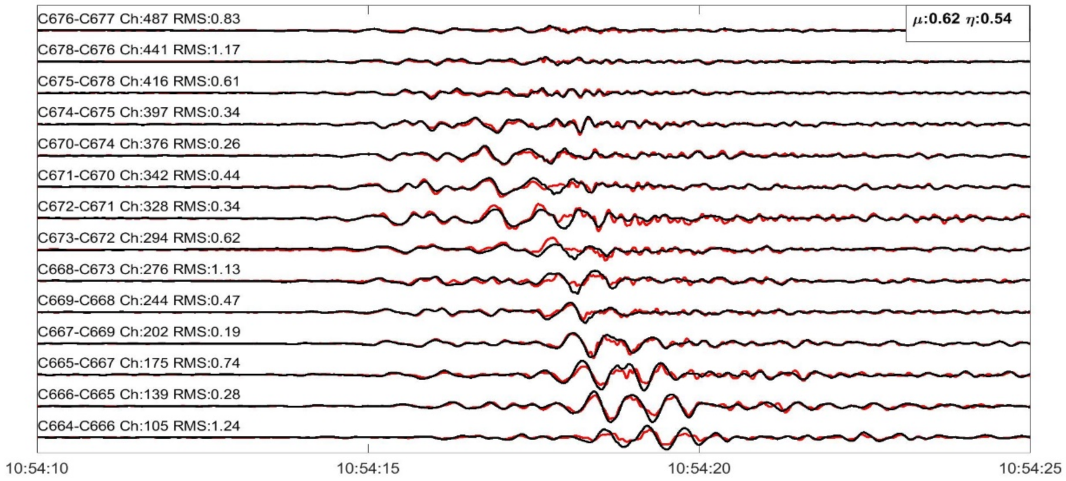

c.

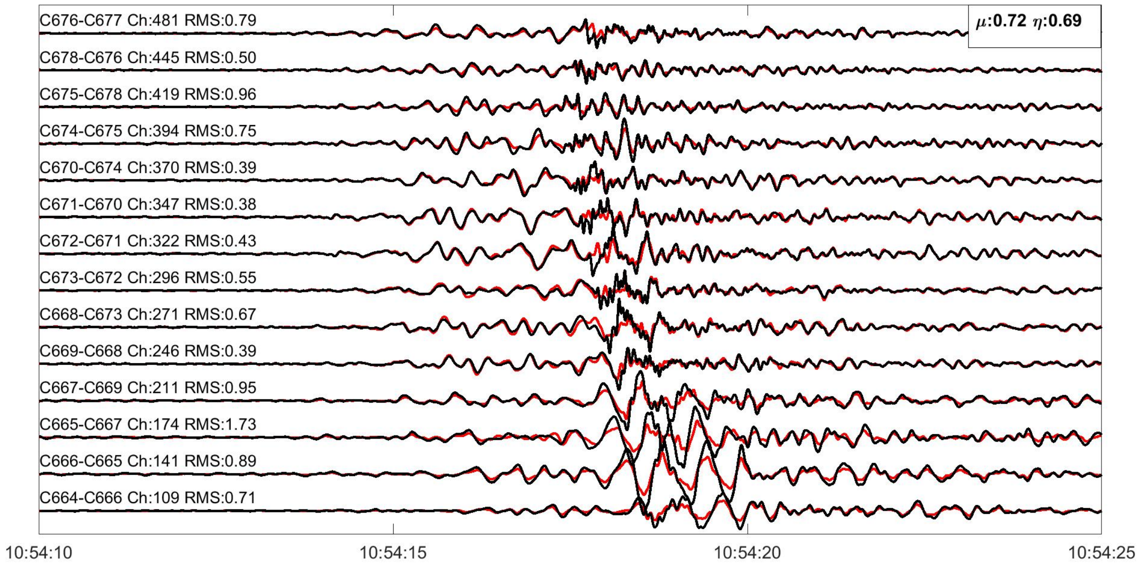

d.

Supplementary Fig. 4 (continued) | c. for adjacent geophones[2] (Method: DAS strain rate and strain validation, spatial displacement gradient (iii) cross-correlation), and **d.** over a gauge length[3] (Method: DAS strain rate and strain validation, strain rate over gauge length). For each trace the text indicates the geophone sensor (a.) or couple (b., c. and d.), the phase velocity *c* used (a. and b.), the DAS channel (d.) and the RMS (Root Mean Square) error between the converted strain and the DAS measurement. For each method, we computed the mean μ and the median η of the normalised RMS error over all traces, as indicated in the upper corner of each figure.

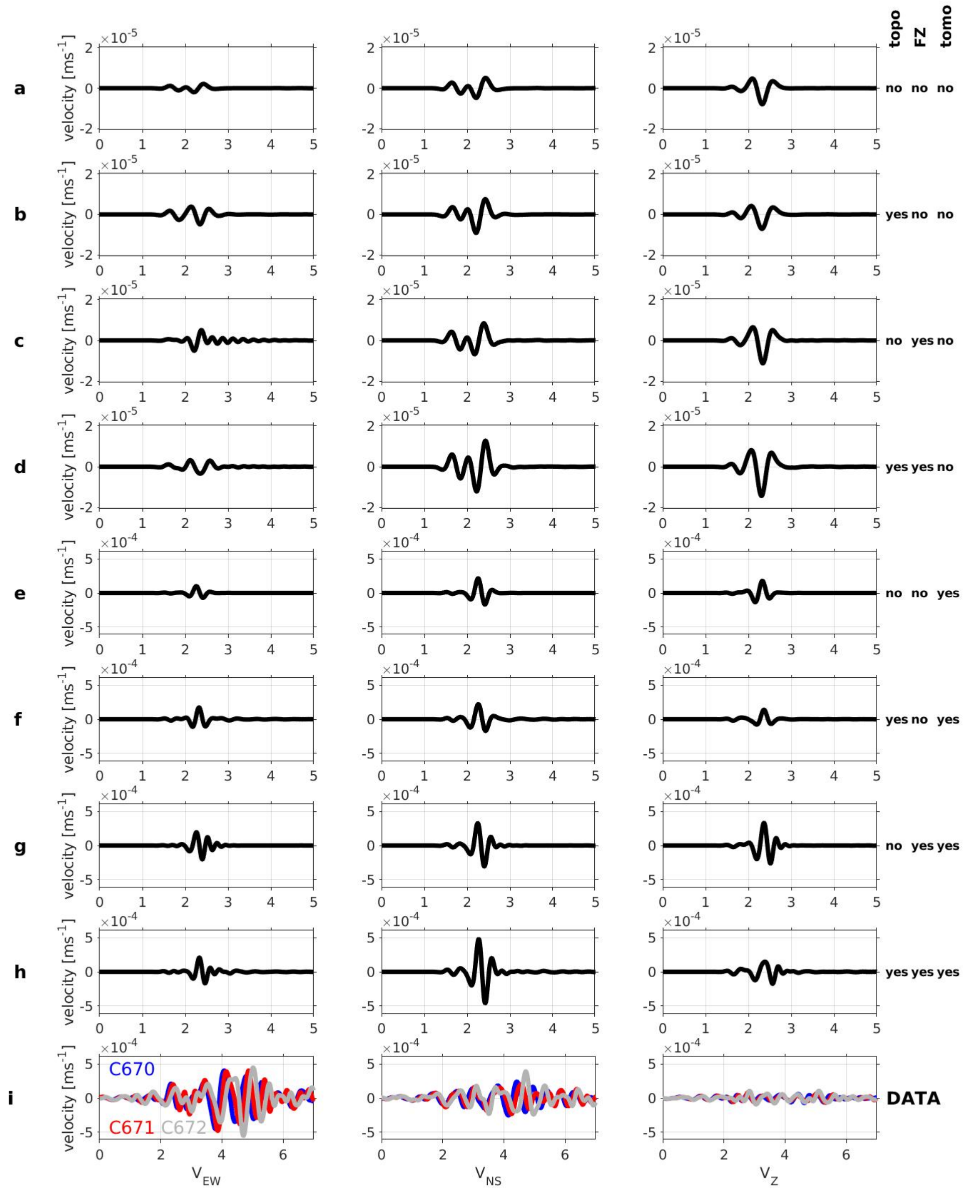

Supplementary Fig. 5 | Simulated ground motions[4] for 9 different models. 3 main features are considered: topography, fault zone and tomographic model with low velocity shallow layers. **a. to h.**: Each line of figures shows synthetic waveforms (with time axis in seconds) for the 3 components (EW, NS and Z) following a specific model in which the 3 features are considered ("yes") or not considered ("no"), as indicated in the text column at the right of the figure. For all models, we performed 3D simulations by triggering a vertical single-force source time function (modelled as a Ricker wavelet with amplitude 1×10^9 N, frequency 2 Hz, and 1.5 s long). **i.** Observed 3-C records (filtered between 0.5 and 5 Hz) from three geophones C670, C671 and C672. C670 and C671 are located nearby the fault zone (Fig. 1), whereas C672 is 70 m further away from the fault zone. Note that the geophone records are quite similar, making it challenging to identify the existence of the fault zone from the geophone records only. This is also confirmed by model waveforms (compare **b.** and **d.**). Models that match qualitatively best observed waveforms include all features (model **h.**). Note that time axis (in seconds) are different in data and models, implying that even slower velocities are required to delay more the modelled waveforms.

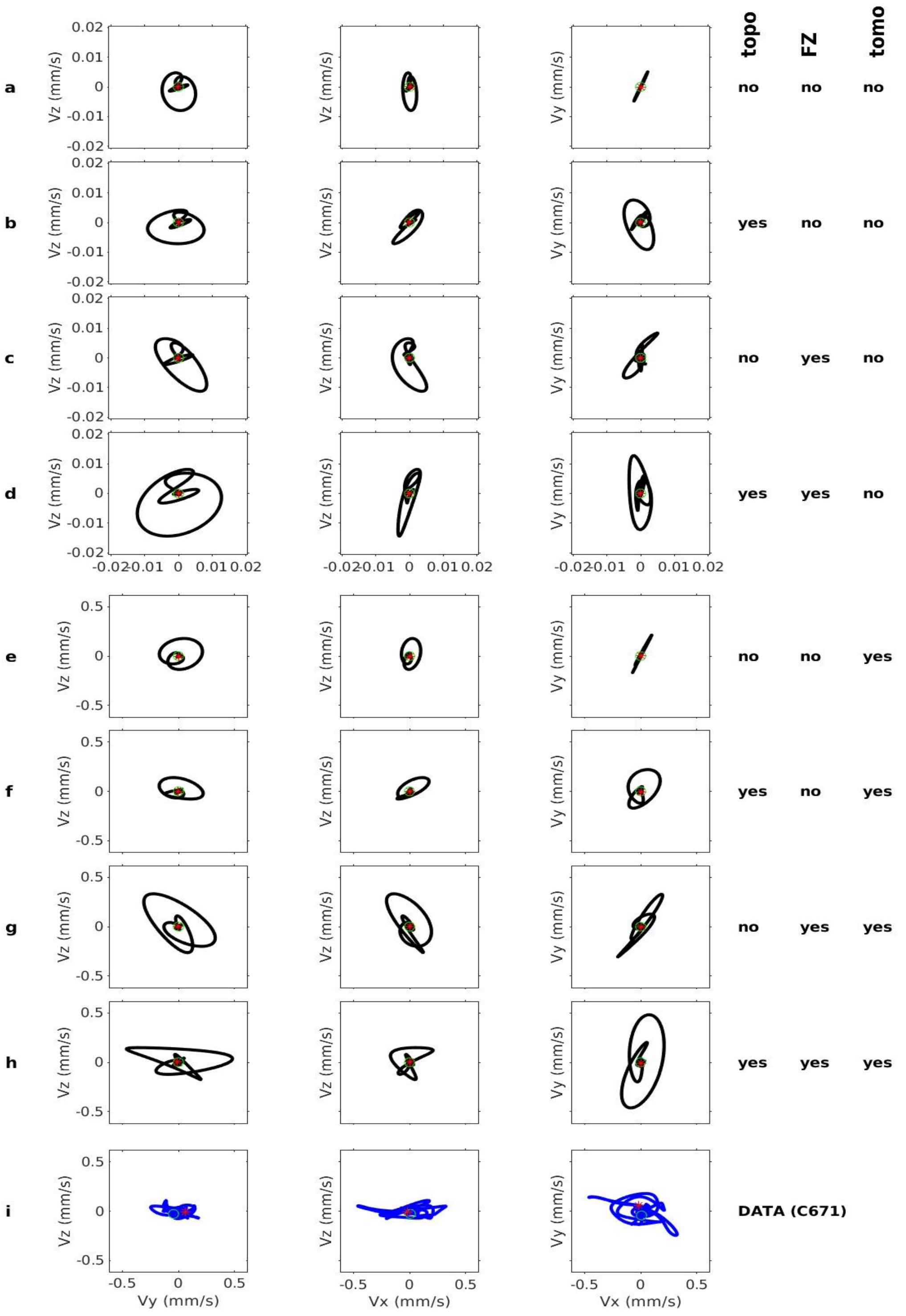

Supplementary Fig. 6 | Simulated particle motions (3 orthogonal planes) at geophone C671 (near a fault zone) for different models. Modelling parameters same as in Supplementary Fig. 5. The model where amplitudes and travel times resembles best data is **h**, where the three features are included.

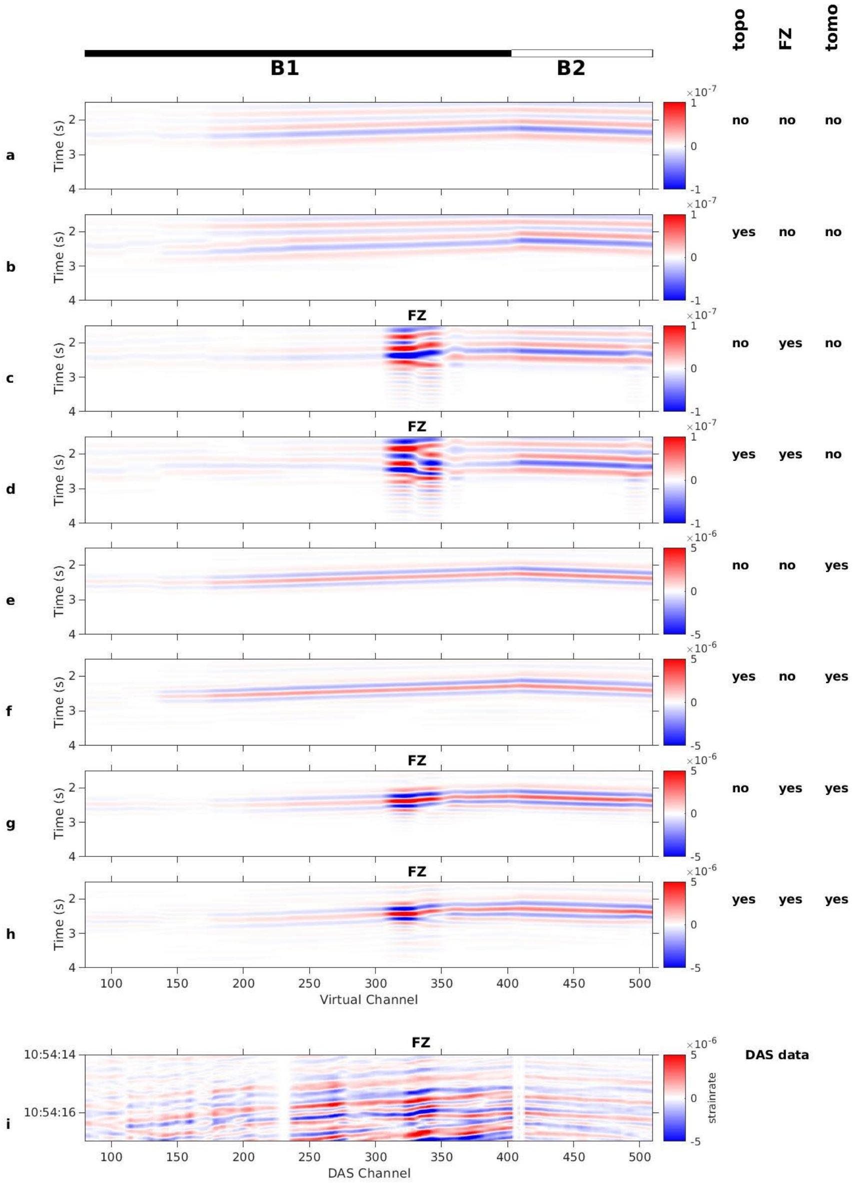

Supplementary Fig. 7 | Simulated strain rate along the virtual fibre. Modelling parameters same as in Supplementary Fig. 5. As DAS sensitivity is higher along the fibre direction, we expect larger strain-rate amplitudes along B2 than along B1. Instead, larger amplitudes occur along branch B1, as seen in both velocity and strain data (e.g., Fig. 2 and 3). Models including the fault zone (FZ) explain increased amplitudes in the fault zone (c., d., g. and h.). Models including tomography and a shallow velocity layer best explain relative strain rate amplitudes between B1 and B2 and travel times (compare b. and f.). Models with topography lead to increase strain rate amplitudes along B1 and decrease those along B2 (compare e. and f; g. and h.). Note that strain rates highlight the fault zone much better than the velocity counterpart, as strain rate highlights horizontal velocity gradient contrasts (spatial gradients), than velocity. The spatial extension of the signal in our figures is also slightly too much enhanced by the spatial interpolation and smoothing, due to the lower resolution of the spatial grid in the modelling (30 m) with respect to the fibre resolution (10 m). Even if not matching perfectly with the observations, those models illustrate the relative contribution of structural features, topography and tomography in the synthetic models.

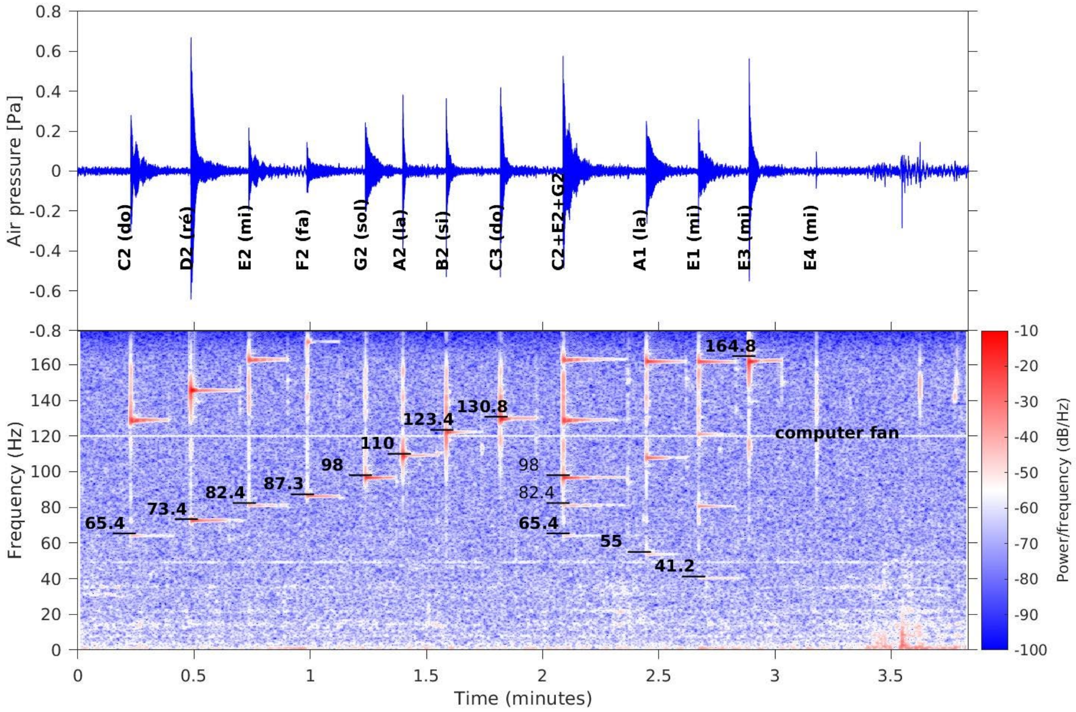

Supplementary Fig. 8 | Infrasound signal high frequency validation with a piano. When a tone is played, the fundamental excites harmonics, which may have larger amplitude than the fundamental[5]. The harmonics vary depending on the instrument, and are integer multiples of the fundamental. This gives the timbre of the instrument. The following sequence was generated (Supplementary Movie 2) by stroking 13 piano keys successively within ~4 minutes. Piano keys played/time after start of video: C2 (do)/0:09; D2 (ré)/0:25; E2 (mi)/0:40; F2 (fa)/0:55; G2 (sol)/1:10; A2 (la)/1:20; B2 (si)/1:31; C3 (do)/1:45; C2+E2+G2 (do/mi/sol)/2:01; A1 (la)/2:22; E1 (mi)/2:36; E3 (mi)/2:49; E4 (mi)/3:06. Note a sustained resonance at about 125 Hz corresponding to noise generated by the cooling system fan of a desktop computer located nearby.

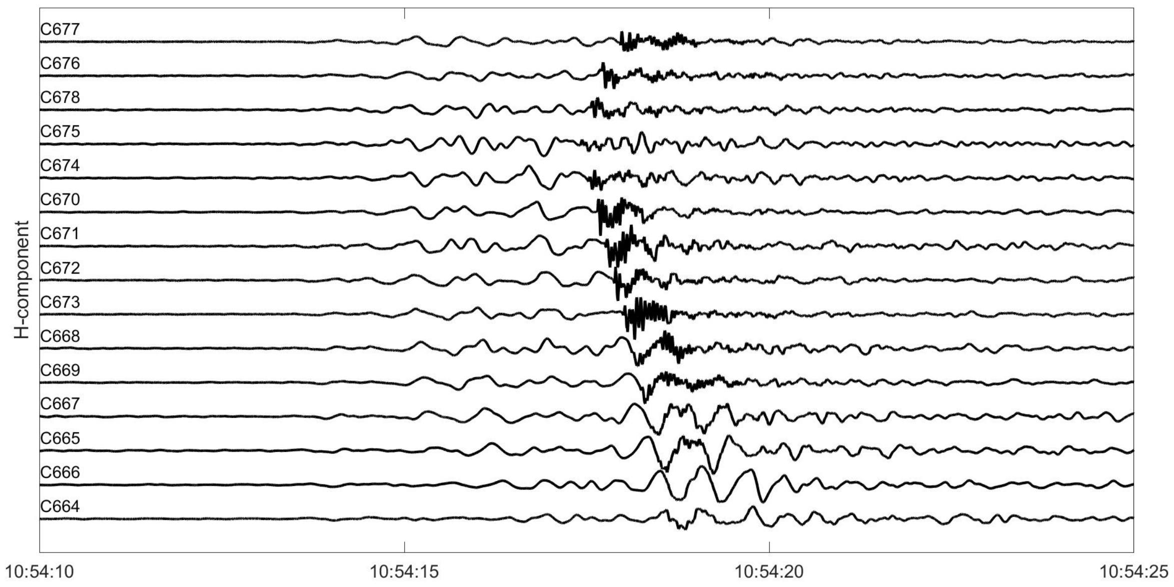

a.

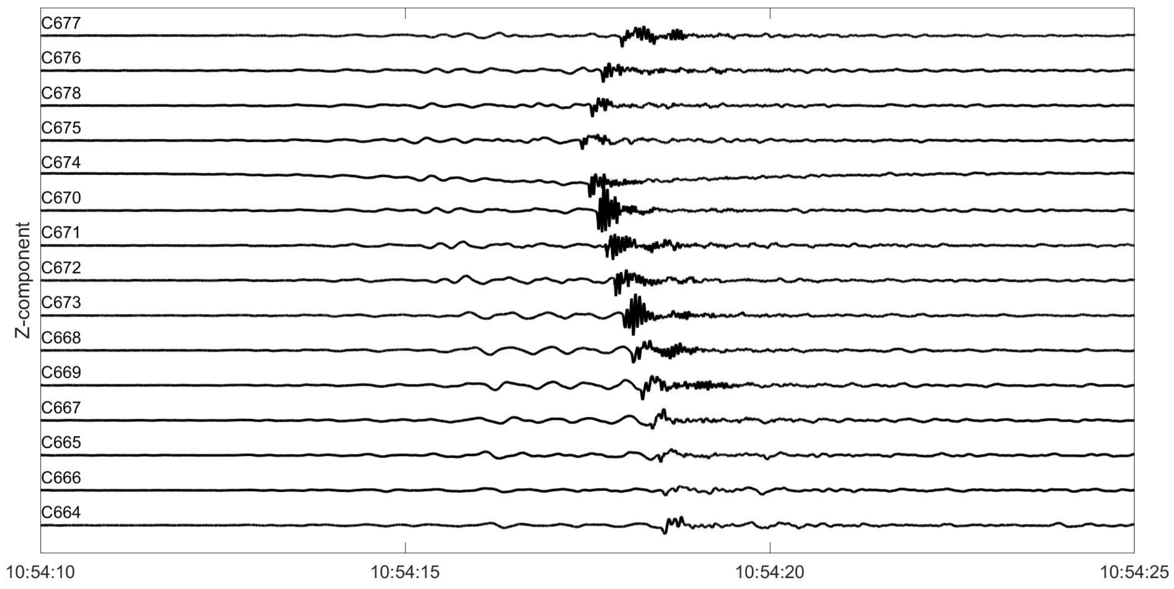

b.

Supplementary Fig. 9 | Geophone records of the explosion at NSEC at 10:54:11. a. Horizontal (H) component projected along the fibre direction (Fig. 1). **b.** Vertical (Z) component.

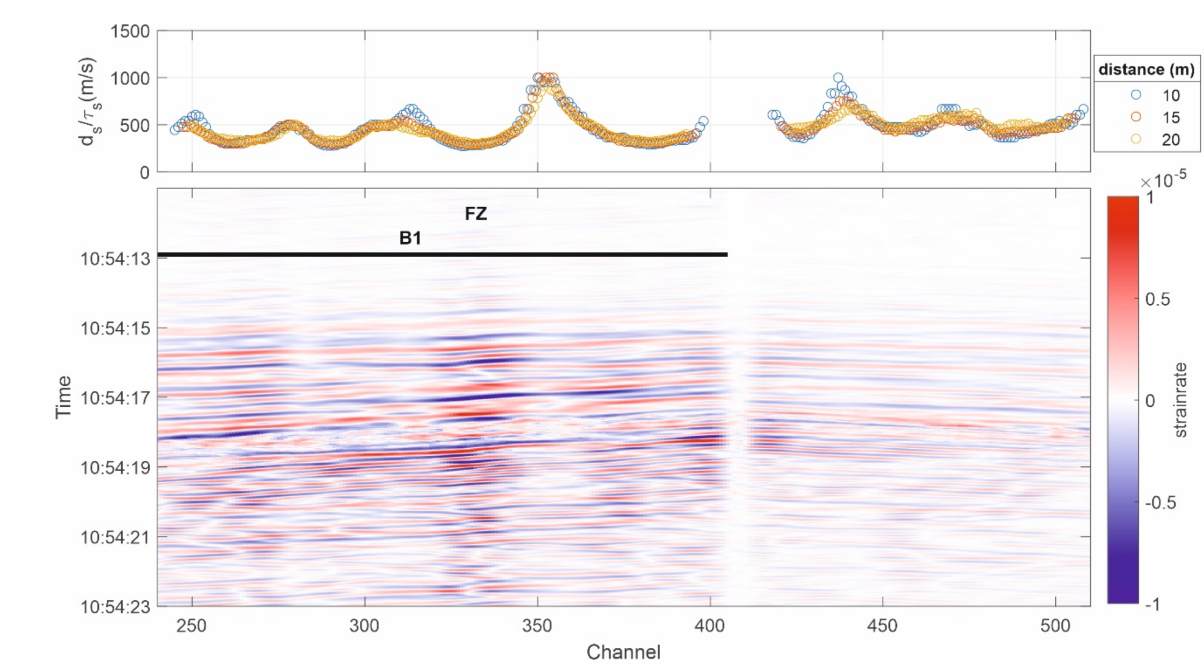

Supplementary Fig. 10 | Ground velocity estimation (top) Profile of the apparent seismic propagation velocity (defined by the ratio of the distance between sensors, d_S and the observed travel time τ_S) along the two linear branches B1 (channels 240 to 400) and B2 (channels 410 to 510) computed for the volcanic explosion on 5 September 2018 (Fig. 3a). Estimates are derived every 2 m using channel couples distant by 10, 15 or 20 m. They yield similar values for the velocities at most locations along the cable. **(bottom)** enhanced seismic signal after wave field separation (Method: Coherent wavefield separation and data enhancement).

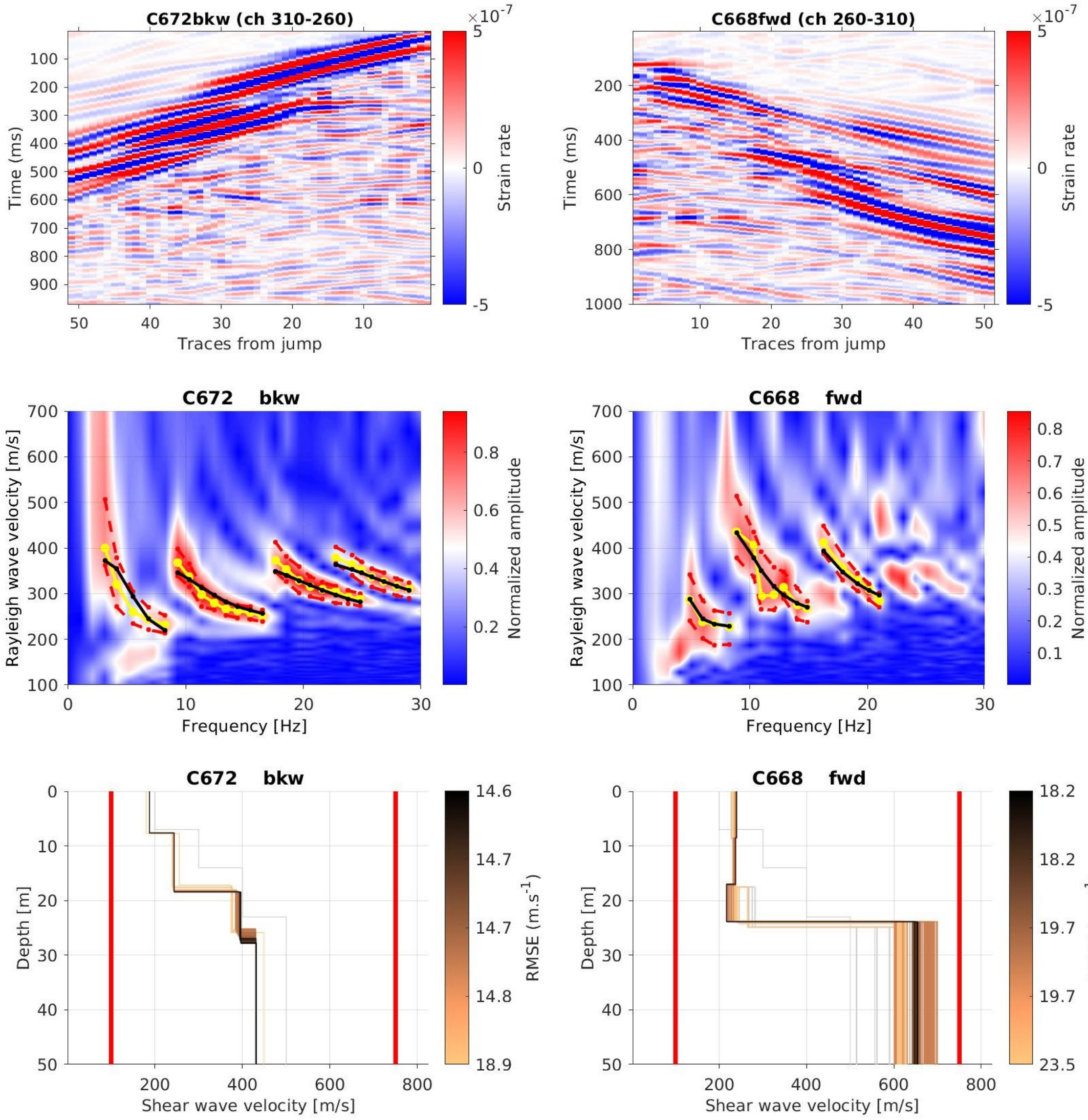

a.

Supplementary Fig. 11 | Multichannel Analysis of Surface Waves (MASW) and Markov chain Monte Carlo inversion of dispersion curves from DAS records. Graphs represent analysis results for 4 jumps performed at locations given by each subfigure title (Fig. 1 for locations). Each subfigure shows the analysis results for several profiles that partly overlap each other. We define a forward branch (fwd) signals for channel numbers larger than the channel of the jump, and a backward branch (bkw) signals for channel numbers smaller than the channel of the jump. The fwd and bkw records are both used separately for the dispersion curve analysis (Supplementary Fig. 12. and Method: Ground velocity estimations, MASW). **(top subplots)** Enhanced strain signals (Method: Coherent wavefield enhancement and separation).

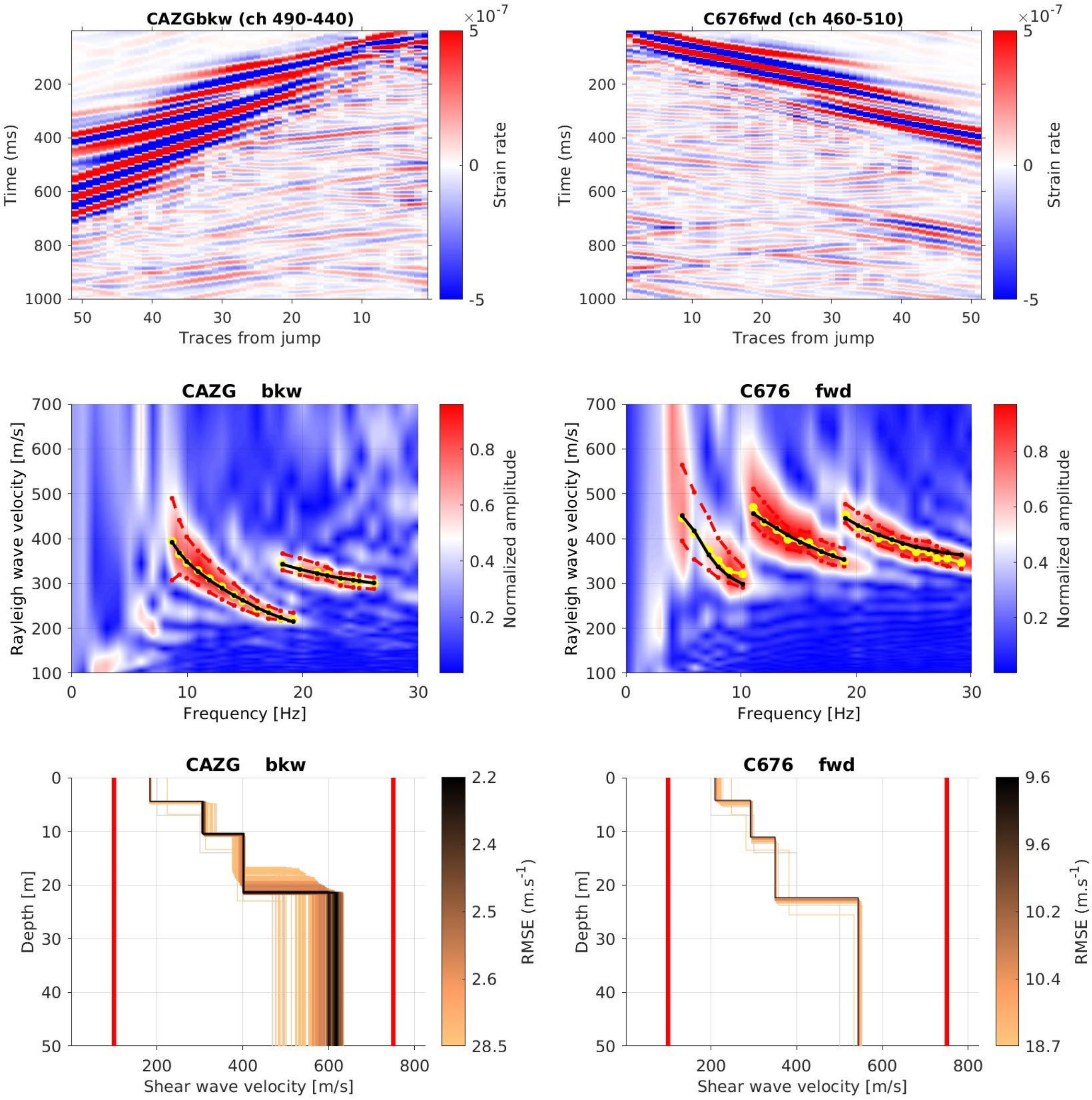

b.

Supplementary Fig. 11 (continued) | (middle subplots) Dispersion spectra in phase velocity/frequency domain. Green dotted lines are the picked dispersion values with their corresponding uncertainty (red dotted lines). Black dotted lines represent the multimode dispersion curves computed using the best inverted model causing the lowest root mean square error (RMSE). **(bottom subplots)** 1D wave velocity retrieved after Markov chain Monte Carlo inversion. Red lines indicate limits of tested models during the inversion. **a.** In contrast to the backward branch at C672, the forward branch at C668 crosses a major structure in the form of a fault zone causing a more complicated Rayleigh wave field (Fig. 1). **b.** Same as in a., but for jumps performed near C676 (forward) and CAZG (backward).

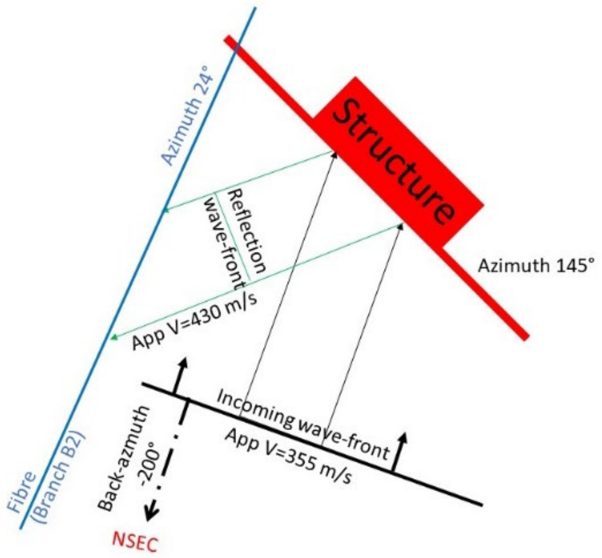

a.

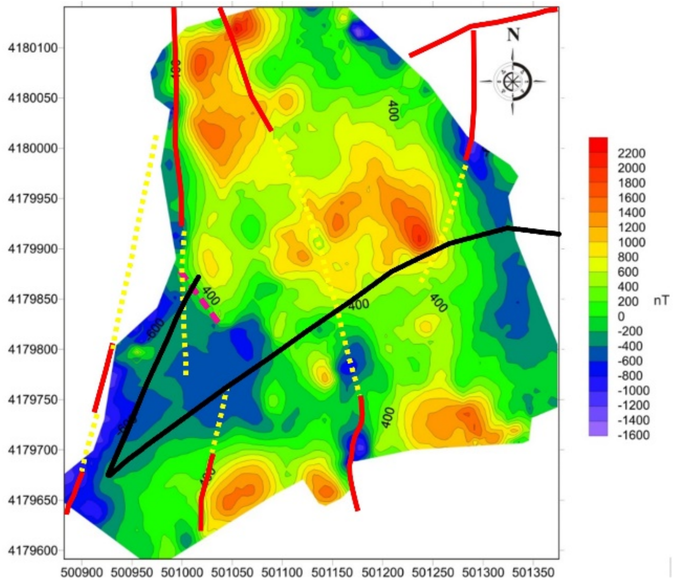

b.

Supplementary Fig. 12 | Interpretation of the negative slowness arrivals from the infrasound induced seismic wave reflection.

In Fig. 4, we observe a clear back-propagating signal at the trace ~490 of the fibre. This back-propagating wave is generated from the reflection of the induced infrasound signal on a local planar structure below the scoria layer. **a.** By analysing apparent velocities of the incoming infrasound wave and the back-propagating wave, we derive a possible reflector having an azimuth of ~145°. **b.** Magnetic map[6] of Piano delle Concazze, where a plausible NW-SE reflector (thick red dotted line) corresponds to a sharp contrast in magnetisation of the subsurface, interpreted as the front of a massive lava flow below the scoria layer.

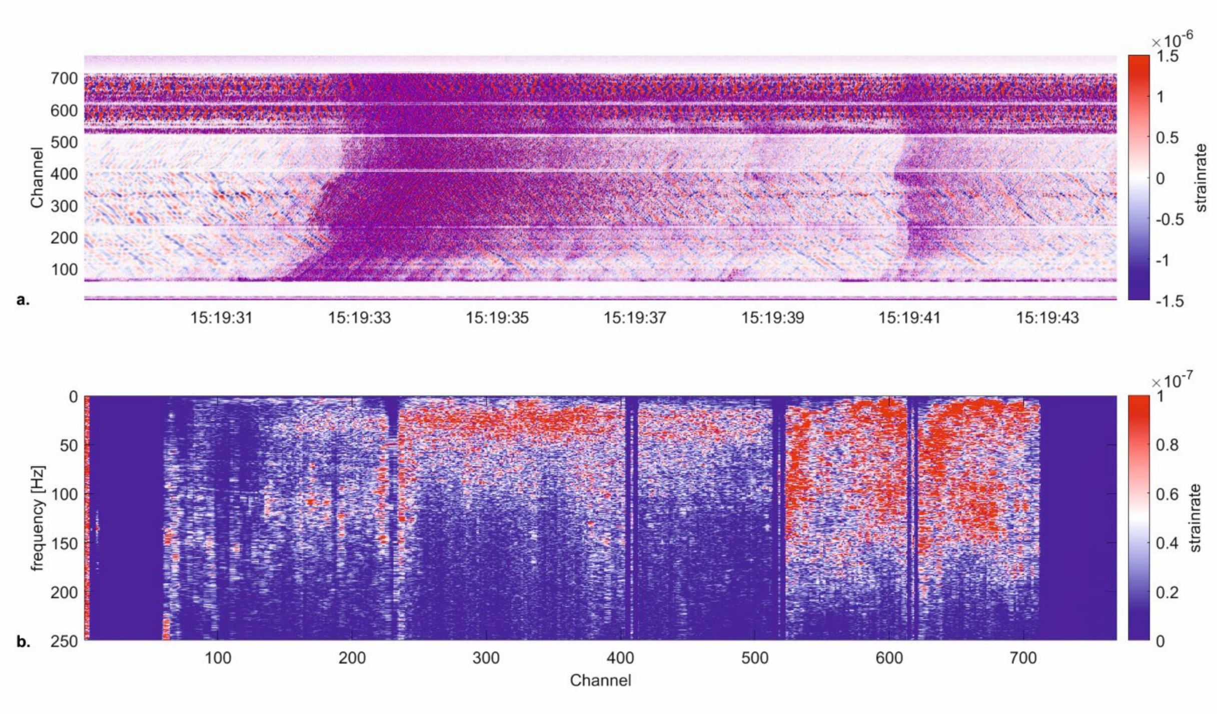

Supplementary Fig. 13 | DAS records and frequency content along the whole fibre of a thunderstorm/lightning event. In addition to the volcanic events described in the paper, we recorded variable meteorological conditions. On 31st August 2018, a thunderstorm with wind gusts, rain and hail occurred at Piano delle Concazze. Those meteorological conditions induced various disturbances in the records, e.g., hail with about 0.5-1 cm diameter fell on the ground, generating an increased high frequency noise especially on the cable at the surface. In addition, lightning struck on 31/08/2018 at about 15:19:31 within 1 km of the observatory. **a.** Acoustic waves recorded with the DAS system (time series). **b.** Frequency analysis along the fibre showing a concentration of spectral energy at about 25-30 Hz, similar to the energy concentrated at those frequencies generated by the volcanic explosion (Fig. 2). Note that, similar to the volcanic explosion, the high-frequency signal is not present on the crater rim (channels 40-150). Channels 510-720 belong to the part of the cable not yet covered with scoriae.

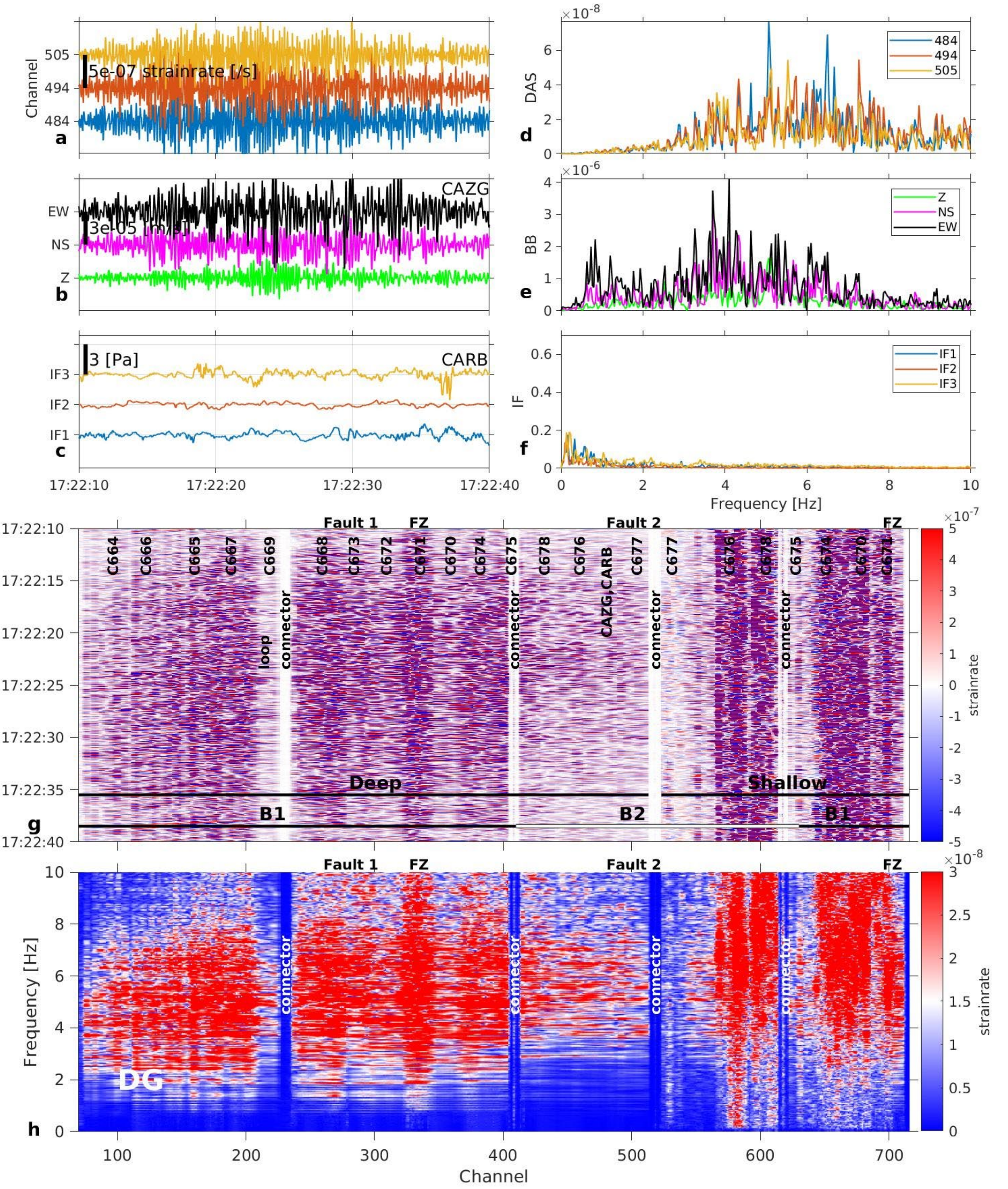

Supplementary Fig. 14 | Transient signals hidden in the tremor: Degassing event (DG).
Filter is 0.1-10 Hz;

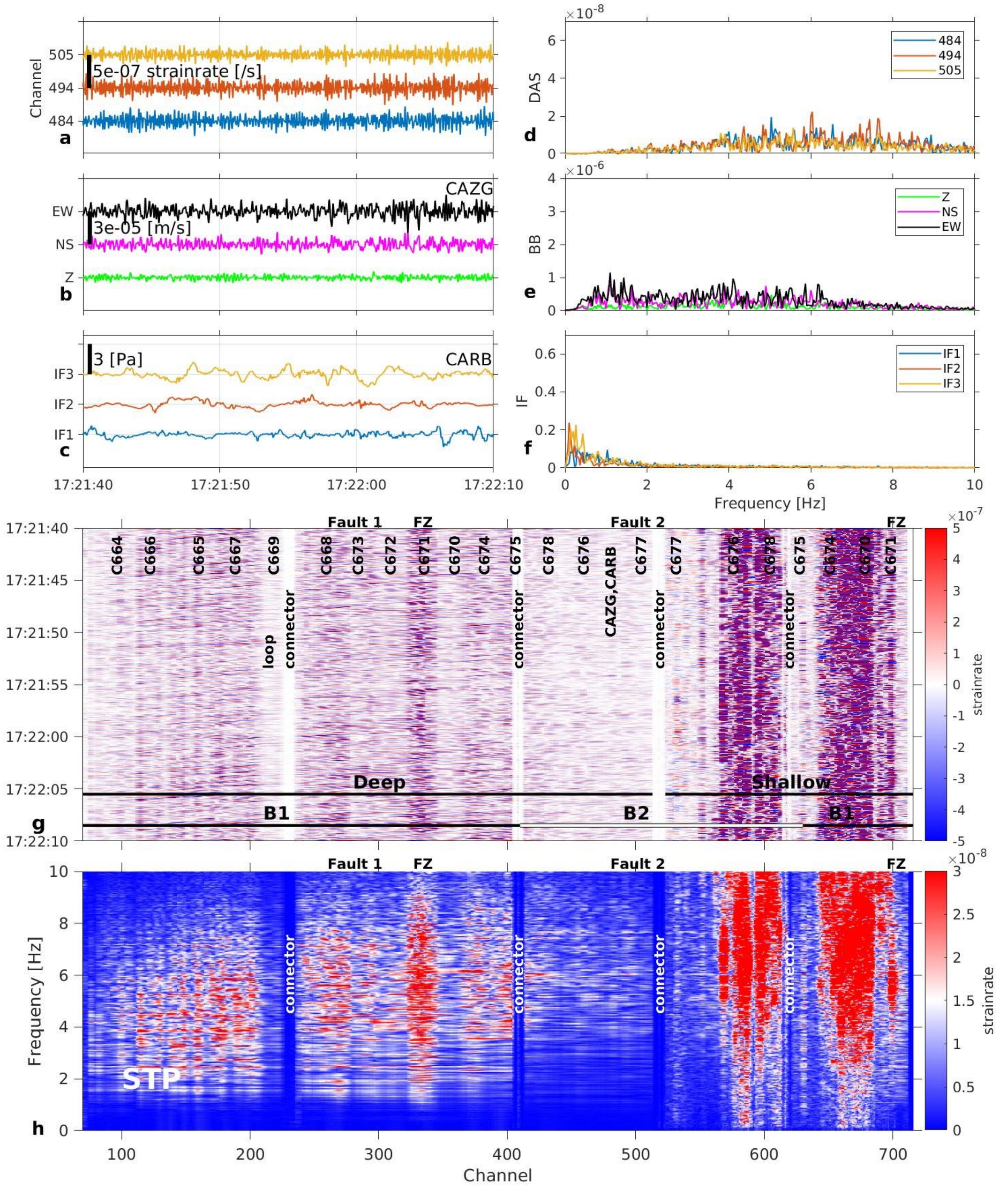

Supplementary Fig. 15 | Transient signals hidden in the tremor: Single Tremor Pulse (STP).
Filter is 0.1-10 Hz

Supplementary Tables

Date	Event Time (UTC)	Crater	Type
9/5/2018	4:16:00	NSEC	Explosion
9/5/2018	6:36:00	NSEC	Explosion
9/5/2018	10:54:00	NSEC	Explosion
9/5/2018	20:12:00	NSEC	Explosion
9/6/2018	21:56:00	NSEC	Explosion
9/7/2018	3:18:00	NSEC	Explosion
9/7/2018	21:56:00	NSEC	Explosion
9/7/2018	23:50:00	NSEC	Explosion
9/8/2018	8:25:00	NSEC	Explosion
9/9/2018	4:55:00	NSEC	Explosion
9/13/2018	6:43:00	NSEC	Explosion

Supplementary Table 1 | Main volcanic events at Etna reported by INGV (http://www.ct.ingv.it/index.php/monitoraggio-e-sorveglianza/prodotti-del-monitoraggio/bollettini-settimanali-multidisciplinari) from 30 August to 16 September 2018. NSEC: New South East Crater (Fig. 1).

Date	Event Time (UTC)	ML	Depth	Area
12/09/2018	09:23	2.2	3.5	2.0 km NE from Ragalna
12/09/2018	14:10	2.0	3.5	2.4 km E from Ragalna
12/09/2018	22:38	2.5	3.8	0.4 km SE from Mount Frumento Supino

Supplementary Table 2 | Main local seismic events (Ml>=2) reported by INGV (http://www.ct.ingv.it/index.php/monitoraggio-e-sorveglianza/prodotti-del-monitoraggio/bollettini-settimanali-multidisciplinari) from 30 August to 16 September 2018.

Sensor #	Type	Longitude °E	Latitude °N	Elevation (m)	DAS Channel
C664	Geophone PE6/B	15.01636	37.76629	2815	96
C665	Geophone PE6/B	15.01503	37.76661	2796	160
C666	Geophone PE6/B	15.01588	37.76653	2812	122
C667	Geophone PE6/B	15.01437	37.76647	2794	188
C668	Geophone PE6/B	15.01325	37.76595	2797	259
C669	Geophone PE6/B	15.01372	37.76622	2795	218
C670	Geophone PE6/B	15.01138	37.76491	2803	359
C671	Geophone PE6/B	15.01183	37.76517	2800	335
C672	Geophone PE6/B	15.01231	37.76543	2797	309
C673	Geophone PE6/B	15.01277	37.76569	2797	284
C674	Geophone PE6/B	15.01093	37.76465	2806	382
C675	Geophone PE6/B	15.01053	37.76439	2805	406
C676	Geophone PE6/B	15.01095	37.76522	2803	459
C677	Geophone PE6/B	15.01138	37.76596	2797	504
C678	Geophone PE6/B	15.01073	37.76479	2805	432
CAZG	Trillium Compact	15.01119	37.76563	2800	484
OEM04	CMG3-ESPC	15.01050	37.76438	2806	407
OEM08	CMG3-ESPC	15.01152	37.76616	2802	517
OEM09	CMG3-ESPC	15.01311	37.76473	2801	-
OEM11	CMG3-ESPC	15.01371	37.76622	2796	218
C725-IF1	BSU	15.01054	37.76440	2808	405
C725-IF2	BSU	15.01071	37.76452	2806	493
C725-IF3	BSU	15.01089	37.76462	2805	384
CARB-IF1	BSU	15.01120	37.76561	2799	484
CARB-IF2	BSU	15.01129	37.76578	2798	494
CARB-IF3	BSU	15.01139	37.76593	2798	505

Supplementary Table 3 | Positions of geophones, broadband seismometers and infrasound sensors, deployed in Piano delle Concazze (Fig. 1). Also shown is the corresponding DAS channel number.

Supplementary Notes

Supplementary Note 1 | Volcano-seismology

Volcano-seismology is the study of seismic and acoustic signals associated with volcanic activity. Its goal is to acquire, analyse and model ground motion waveforms on active volcanic settings[7], in order to derive characteristics of volcano structures and infer the nature of the sources and mechanisms generating those signals[8], in association with structural features, magma migration and related hydrothermal fluids processes, and contribute to forecast the volcanic behaviour prior and during eruptions.

Volcanic processes generate many signals and waveforms with various amplitude and frequency content[9].

Volcano-tectonic (VT) events can be connected with fracturing processes of rock under stress due to magma or fluid migration. Those are the most common events and are early precursors for increased activity in a volcano[10]. They are often similar to tectonic earthquakes (frequencies 1-25 Hz), and also have similarities with induced earthquakes in exploited geothermal areas or oil and gas reservoir settings.

Long-Period (LP) events (also called Low-Frequency) are generally thought to be associated with resonance or transport of fluids within the conduit and its surrounding[11], but other explanations have been proposed[12]. Their frequency content range 0.2 to 5 Hz generally. In geothermal systems they have been rarely observed, where they may have higher frequencies. In volcanic settings, they often precede and accompany volcanic eruptions[8].

Very-Long Period (VLP) events are associated with inertial movement of heat, gas and magma movement, and interactions between magma and subterranean water within the volcanic plumbing system[7]. Their frequency content is below 0.2 Hz. LP and VLP do not occur at all volcanoes. Understanding the source mechanisms of LP and VLP signals is a key component in assessing volcano unrest and providing early warning for ensuing eruptions[13].

Tremor is one of the most enigmatic signals recorded on volcanoes. Tremor signals are usually of long duration (several tens of seconds to months) and may be dominated by one or more sharp frequency peaks; tremor can be sometimes harmonic with one single frequency around 1 to 4 Hz. At several volcanoes, tremor and LP signals share the same frequency band and seem to be generated from similar source processes. One of the main differences between them resides in their duration. LP signals may last up to 1 minute, whereas tremor can last for minutes to months. It is sometimes held that tremor is a series of low-frequency events occurring at intervals of a few seconds, but also linked to fluid pulses migrating in fractures. Tremor is associated with all volcanic eruptions and its increasing amplitude is often an eruption precursor[13]. Although different models have been proposed to explain the source mechanism of tremor[12], most researchers concur in attributing its origin to the complex interplay between magmatic-hydrothermal fluids and their hosting rocks.

Volcanic explosions are due to sudden expansion of pressurized volcanic gases at or close to the ground surface. Explosions produce both seismic waves propagating in the ground and also acoustic pressure signals propagating in the atmosphere, first as supersonic shock waves then as sonic acoustic waves[14]. Acoustic waves decay with the inverse of the travelled distance due to geometrical spreading, a model which is not completely true close to the

source. The identification of the transition between supersonic and sonic propagation has been used to estimate properly source parameters of volcanic explosions[15]. Source mechanisms of infrasound signals have been related to (i) the acoustic resonance of magma in the conduit, triggered by explosive sources, (ii) local bubble coalescence within a foam, and (iii) Strombolian bubble vibration.

Other signals can be recorded in association to other volcanic phenomena, such as lahars, rock falls. The terminology given here varies from volcano to volcano, and a complete review can be found in many references[7, 8,14,16].

Supplementary Note 2 | Etna volcano information

Mount Etna (3300 m) is the largest active volcano in Europe, where many eruptions may occur per year. Main hazards at Etna volcano are associated with volcanic activity comprising ash fallout, lava flow, earthquakes and sector collapses. The largest historical eruptions reached the city of Catania in 1669 and many other villages around the volcano. The population living under the threat of Etna volcanic activity counts over 1 million. Etna has therefore been the subject of many research projects. The tectonic and geodynamical context explain the global features of Etna structure and activity. A focus has been offshore investigations in the Ionian Sea[17]. The structure of Etna has been studied from multidisciplinary investigations[18,19,20,21,22,23]. Contributions from seismology at Etna range from tomographic imaging using high-density networks[19,21] to the deployment of seismic arrays that allow high-resolution tracking of volcanic activity[22,23,24]. Volcano seismic signals at Etna take a variety of forms[23,24,25,26,27,28,29,30,31,32,33]. Many studies have focused on the nature of LP, tremor, explosion signals with volcano-seismology methods. Long Period (LP) events also called Low Frequency (LF) events at Etna, deliver energy at frequencies ranging from 0.5 to 5 Hz[24,34]. An alternative model for the generation of LP events at Etna has been put forward[12], in which LP signals can also be generated in a dry medium, implying that they may not be direct indicators of fluid migration. However, in low-viscosity basaltic magmatic systems such as those found at Etna, LP signals have been linked to trigger mechanisms associated with unsteady transport of gases and magma through geometrically complex feeding systems[7,24]. Volcanic tremor at Etna volcano is a continuous seismic signal at 1 to 2 Hz which is generally observed in association with magmatic and hydrothermal activity[24,31,32]. Finally, explosions are often recorded and studied with infrasound and seismologic records[15,26,27,28,35]. INGV (www.ct.ingv.it) reports seismic and volcanic activity at Etna, such as earthquakes, tremor, explosive events based on those definitions. During our experiment (30 August- 16 September 2018), no significant seismicity was reported: only three local earthquakes with magnitude ML >=2.0 occurred in the Etnean area (Supplementary Table 1). In early September, weak strombolian activity, episodic explosive events (Supplementary Video 1) and small degassing pulses (Supplementary Video 2) occurred at the summit craters (Supplementary Table 2; http://www.ct.ingv.it/index.php/monitoraggio-e-sorveglianza/prodotti-del-monitoraggio/bollettini-settimanali-multidisciplinari). The level of volcanic tremor was also low, with a small increase on 14 September.

Supplementary Note 3 | High frequencies and infrasound sensor: case study with a piano

Infrasound sensors did not show frequencies higher than 5 Hz, whereas seismic stations and DAS measurements recorded frequencies up to more than 20 Hz, associated with the 5 September 2018 explosion (Fig. 2). Are our infrasound sensors able to measure frequencies of 16 Hz and above at all? In order to test the frequency range and the sensitivity of the infrasound sensors, we performed a test in the music hall. We successively generated a series of selected frequencies (audible low frequency sounds) with a piano[5]. We recorded the generated pressure waves with the same array of infrasound sensors (put together on a plate) at a distance of about 1.5 m from the piano. Data was collected with a Cube datalogger with a sampling frequency of 400 Hz. Supplementary Fig. 8 shows the infrasound record and the associated spectrogram. Frequencies associated to each note are roughly in accordance with the expected frequency (note: we could have used the infrasound sensors to tune the piano). This small experiment confirms that the infrasound sensors are able to record frequencies up to at least 200 Hz. This strongly suggests that the high frequencies observations at Etna volcano cannot be identified with the atmospheric pressure wave of the explosion. We conclude that the high frequencies are generated in the ground, where they are sensed by the fibre and the seismometers.

Supplementary Note 4 | Coupling between cable and scoriae

Cable deployment schedule

Coupling of fibre optic cables with ground media is the subject of many discussions[36,37,38]. We deployed the cable in a trench at about 15-25 cm depth. As the material is uniform, we expect uniform coupling along the cable. We extended the length of the buried cable with additional cable, that we laid simply at the ground surface on the scoriae. After several days, on 1st September 2018, we buried the superficial cable under few centimetres of scoria only (Supplementary Fig. 1).

Cable properties

We used a single loose tube telecommunication fiber optic cable (SLO), in which 12 multimode optical fibers are embedded in gel and surrounded by a loose tube made of thermoplastic material (PBT) with an outer diameter of 2.7 mm and a wall thickness of 0.35 mm. As dielectric armour and rodent protection, a 2400 tex glass yarn was used and surrounded by a 7 mm diameter polyethylene outer jacket with a wall thickness of 1.4 mm. The stretch of the cable is 0.3% at 190 N, 0.5% at 350 N and 1% at 580 N.

Scoria properties

We measured the density and the angle of repose of the scoriae material in the laboratory. As unconsolidated volcanic ash, the scoriae have a dry bulk density of ~520+/-10 $kg.m^{-3}$. As an attempt to investigate the slope stability of the material, we poured scoriae on a flat surface and measured the height and diameter of the cone, from which the angle of repose is found to be 33.7° and 35.8° in dry and wet conditions, respectively.

Cable response analysis

Our observations suggest that the coupling of the cable with the ground can be sufficient for a cable covered with just a few centimetres of scoriae (Supplementary Fig. 1). For the explosion shown in Fig. 2 a maximum strain amplitude of $1.5x10^{-6}$ (strain) was computed from the integration of the strain rate at the position of the fault zone. Based on a model of a fully elastic and perfectly bonded multilayer, radial symmetric cable design, where stress is uniformly applied to the outer layer of the cable, the response of the cable to external strain, as well as the resulting shear forces at individual interfaces within the multilayer cable, can be calculated[38]. It is assumed[38] that the gel can be approximated with a shear thinning Bingham fluid. According to the specifications of the manufacturer, viscosity information is given for shear rates of 50 and 200 s^{-1}. Based on the Bingham model, a yield point of 60 Pa was estimated for the gel. Below, the gel is assumed to behave like an elastic medium. For a 10 m cable segment and neglecting creep, we conclude that the required force to stretch the cable by 10^{-6} strain is below 1 N. For a force of 1 N acting on the surface of the cable, shear forces of less than 1.3 and 0.2 Pa were calculated at the interface fibre-gel and gel-tube, respectively. These forces are 1-2 orders of magnitude lower than the yield strength of the gel. Hence, we can conclude that for strain values recorded during the experiment, the cable behaves fully elastically, despite the gel surrounding the fibre.

For a much stiffer cable buried in sand[38], the force needed to be applied at the cable-granular material interface in order to stretch the cable is much lower than the force needed to reorganize the granular material itself. We parametrize the model with our cable and the scoria properties. As a first approximation, the friction angle is similar to the angle of repose and there is continuous contact between cable and scoria grains. In those conditions, the reorganization of the scoriae needs significantly higher strain amplitudes to force a grain reorganization. The estimated shear forces at the cable-scoria interface to reach the Mohr-Coulomb failure criteria for the scoriae are about two orders of magnitude higher than the forces needed to stretch the cable by 10^{-6} strain on a 10 m segment. Even if a continuous contact is not given, higher point forces at the contact locations between cable and scoriae are assumed to provide sufficient contact force to prevent slip between cable and grains.

Supplementary references

1. Lindsey, N.J., Dawe, C. T. and Ajo-Franklin, J.B. On the Broadband Instrument Response of Fiber-Optic DAS Arrays. *Journal of Geophysical Research* **125**, 2, doi:10.1029/2019JB018145 (2020).

2. Jousset, P. & Rohmer, J. Evidence of remotely triggered micro-earthquakes during salt cavern collapse. *Geophysical Journal International* **191**, 1, 207-223 (2012).

3. Wang, H., X. Zeng, D. E., Miller, D., Fratta, K. L., Feigl et al. Ground motion response to an ML4.3 earthquake using co-located distributed acoustic sensing and seismometer arrays. *Geophysical Journal International* **213**,2020–2036. doi: 10.1093/gji/ggy102 (2018).

4. Jousset, P., Neuberg, J. & Jolly, A. Modelling low-frequency volcanic earthquakes in a viscoelastic medium with topography. *Geophysical Journal International* **159** (2), 776-802 (2004).

5. (92). Dunne, E. & M., McConnel. Pianos and Continued Fractions. *Mathematics Magazine* **72**, 2, 104-115 (1999).

6. Napoli, R., Currenti, G, & Sicali, A. Magnetic signatures of subsurface faults on the northern upper flank of Mt Etna (Italy). *Annals of Geophysics* **64,** 1, PE108, doi:10.4401/ag-8582 (2021).

7. Chouet, B. & Matoza, R.S. A multi-decadal view of seismic methods for detecting precursors of magma movement and eruption. *Journal of Volcanology Geothermal Research* **252**, 108-175, https://doi.org/10.1016/j.jvolgeores.2012.11.013 (2013).

8. McNutt, S.R. & Roman, D. C. Volcanic Seismicity, in H. Sigurdsson (Ed.), *The Encyclopedia of Volcanoes (2nd Ed.),* Academic Press, https://doi.org/10.1016/B978-0-12-385938-9.00059-6 (2015).

9. Pallister, J. & S. R., McNutt. Synthesis of volcano monitoring, in Encyclopedia of Volcanoes, 2nd ed., edited by H. Sigurdsson et al., Elsevier, Amsterdam, doi:10.1016/b978-0-12-385938-9.00066-3 (2015).

10. Roman, D. C. & Kashman, K. V. The origin of volcano-tectonic earthquake swarms. *Geology* **34**, 6, 457-460 (2006).

11. Neuberg, J., Lucket, R., Ripepe, M., & Braun, T. Highlight from a seismic broadband array on Stromboli. *Geophysical Research Letters* **21**, 9, doi.org/10.1029/94GL00377 (1994).

12. Bean, C. J, L. De Barros, I. Lokmer, J-P Metaxian, G. O'Brien, S. Murphy. Long-Period seismicity in the shallow volcanic edifice formed from slow-rupture earthquakes. *Nature Geoscience* **7**, 71-75, doi:10.1038/ngeo2027 (2014).

13. Zuccarello L., Burton M.R., Saccorotti G., Bean C. J., and Patanè D. The coupling between very long period seismic events, volcanic tremor, and degassing rates at Mount Etna volcano. *Journal of Geophysical Research* **118**, 1-12, doi:10.1002/jgrb.50363 (2013).

14. McNutt S.R., Thompson G., Johnson J., De Angelis S., Fee D. Seismic and Infrasonic Monitoring, Editor(s): in H. Sigurdsson (Ed.), *The Encyclopedia of Volcanoes (2nd Ed.), Academic Press*, ISBN 9780123859389, https://doi.org/10.1016/B978-0-12-385938-9.00063-8 (2015).

15. Medici, E.F., Allen, J.S. & Waite, G.P. Modeling shock waves generated by explosive volcanic eruptions. *Geophysical Research Letters* **41**, 414-421, doi:10.1002/2013GL058240 (2013).

16. Johnson, J. & Ripepe. M. Volcano Infrasound: A Review. *Journal of Volcanology and Geothermal Research* **206**, 3-4 (2011).

17. Gutscher, M-A., Royer, J-Y., Graindorge, D., Murphy, S., Klingelhoefer, F., Aiken, C., Cattaneo, A., Barreca, B., Quetel, L. & Riccobene G., Photoniques 3, 32 (2019).

18. Bonaccorso, A., Calvari, S., Coltelli, M., Del Negro, C., Falsaperla, S. Mount Etna: Volcano laboratory. *Geophysical Monograph AGU* **143**, 384 pp. doi: 10.1029/GM143 - ISBN: 978-1-118-66579-4 (2004).

19. Alparone, S., Barberi, G., Cocina, O., Giampiccolo, E., Musumeci, C., Patanè, D. Intrusive mechanism of the 2008–2009 Mt. Etna eruption: Constraints by tomographic images and stress tensor analysis. *J. Volcanol. Geotherm. Res.* **50–63**. 10.1016/j.jvolgeores.2012.04.001 (2012).

20. Patanè, D., A., Aiuppa, M., Aloisi, B., Behncke, A., Cannata, M., Coltelli, G., Di Grazia, S., Gambino, S., Gurrieri, M., Mattia, G., Salerno. Insights into magma and fluid transfer at Mount Etna by a multiparametric approach: A model of the events leading to the 2011 eruptive cycle, *J. Geophys. Res. Solid Earth* **118**, 3519–3539, doi:10.1002/jgrb.50248 (2013).

21. Patanè, D., G. Barberi, O., Cocina, P., De Gori, C., Chiarabba. Time- Resolved Seismic Tomography Detects Magma Intrusions at Mount Etna. *Science* **313**, 821-823 (2006).

22. Barberi, G., Cocina, O., Maiolino, V., Musumeci, C., Privitera, E. Insight into Mt. Etna (Italy) kinematics during the 2002–2003 eruption as inferred from seismic stress and strain tensors. *Geophysical Research Letters* **31**, 21. DOI:10.1029/2004GL020918 (2004).

23. Saccoroti, G., Zuccarello, L., Del Pezzo, E., Ibanez, J. & Gresca, S. Quantitative analysis of the tremor wavefield at Etna volcano, Italy. *Journal of Volcanology and Geothermal Research* **136**, 223-245 (2004).

24. Di Lieto, B., Saccorotti, G., Zuccarello, L., La Rocca, M., Scarpa, R. Continuous tracking of volcanic tremor at Mount Etna, Italy. *Geophysical Journal International* **169**, 699–705, doi: 10.1111/j.1365-246X.2007.03316.x (2007).

25. De Angelis, S., Haney, M.M., Lyons, J.J., Wech, A., Fee, D., Diaz-Moreno, A. and Zuccarello, L. Uncertainty in detection of volcanic activity using infrasound arrays: Examples from Mt. Etna, Italy. *Front. Earth Sci.* **8**, 169. doi: 10.3389/feart.2020.00169 (2020).

26. Diaz- Moreno, A., Lezzi, A.M., Lamb, O. D., Fee, D., Kim, K., Zuccarello, L., De Angelis, S. Volume flow rate estimation for small explosions at Mt. Etna, Italy, from acoustic waveform inversion. *Geophysical Research Letters*, doi:10.10292019GL084598 (2019).

27. Cannata, A., Di Grazia, G., Aliotta, M., Cassisi, C., Montalto, P., Patanè, D. Monitoring Seismo-volcanic and Infrasonic Signals at Volcanoes: Mt. Etna Case Study. *Pure and Applied Geophysics* **170**. 10.1007/s00024-012-0634-x (2013).

28. Cannata, A., Montalto, P., Privitera, E., Russo, G., Gresta, S. Tracking eruptive phenomena by infrasound: May 13, 2008 eruption at Mt. Etna. *Geophysical Research Letters* **36**, 5. DOI:10.1029/2008GL036738 (2009).

29. Lokmer, I., C.J. Bean, G. Saccorotti, D. Patanè. Moment-tensor inversion of LP events recorded on Etna in 2004 using constraints obtained from wave simulation tests. *Geophysical Research Letters* **34**, L22316, doi:10.1029/2007GL031902 (2007).

30. Cannata, A., Di Grazia, G., Montalto, P., Aliotta, M., Patanè, D., Boschi, E. Response of Mount Etna to dynamic stresses from distant earthquakes. *Journal of Geophysical Research* **115**, B12, doi:10.1029/2010JB007487 (2010).

31. Carbone, D., Zuccarello, L., Messina, A., Scollo, S., Rymer, H. Balancing bulk gas accumulation and gas output before and during lava fountaining episodes at Mt. Etna. *Scientific Report* **5**, 18049; doi: 10.1038/srep18049 (2015).

32. Sciotto, M., Cannata, A., Gresta, S., Privitera, E., Spina, L. (2013). Seismic and infrasound signals at Mt. Etna: Modeling the North-East crater conduit and its relation with the 2008–2009 eruption feeding system. *Journal of Volcanology and Geothermal Research* **254**, 53-68. DOI:10.1016/j.jvolgeores.2012.12.024

33. Cannavò, F., Cannata, A., Cassisi, C., Di Grazia, G., Montalto, P., Prestifilippo, M., Privitera, E., Coltelli, M., Gambino, S. A multivariate probabilistic graphical model for real-time volcano monitoring on Mount Etna, Journal of Geophysical Research: *Solid Earth* **122**, 5, 3480-3496. DOI: 10.1002/2016JB013512 (2017).

34. Di Grazia, G., Cannata, A., Montalto, P., Patanè, D., Privitera, E., Zuccarello, L., Boschi, E. A multiparameter approach to volcano monitoring based on 4D analyses of seismo-volcanic and acoustic signals: The 2008 Mt. Etna eruption. *Geophysical Research Letters* **36**, L18307, doi:10.1029/2009GL039567 (2009).

35. Pering, T. D., G., Tamburello, A. J. S., McGonigle, A., Aiuppa, M. R., James, S. J., Lane, M., Sciotto, A., Cannata, and D., Patanè. Dynamics of mild Strombolian activity on Mt. Etna. *Journal of Volcanology and Geothermal Research* **300**, 103–111, doi: 10.1016/j.jvolgeores.2014.12.013 (2015).

36. Zhang, C.C., Zhu, H.H. & B., Zhi. Role of the interface between distributed fibre optic strain sensor and soil in ground deformation measurement. *Scientific Reports* **6**, 36469, doi: 10.1038/sep36469 (2016).

37. Krawczyk, C.M. Wie Glasfaserkabel als Geosensoren zur Erkundung und Überwachung des Untergrunds genutzt werden können – Anwendungen und Potenzial von ortsverteilten faseroptischen Messungen (How fibre optic cables can be used as geosensors to explore and monitor the subsurface – Applications and Potential of distributed acoustic sensing). Brandenburgische Geowiss. *Beiträge* **28** (1/2), 15-28 (2021).

38. Reinsch, T., Thurley, T. & Jousset, P. On the mechanical coupling of a fiber optic cable used for distributed acoustic/vibration sensing applications — a theoretical consideration. *Measurement Science and Technology* **28**, 12. http://doi.org/10.1088/1361-6501/aa8ba4 , (2017).
