# Supplementary material for: Fibre optic distributed acoustic sensing of volcanic events
Source: Nat Commun. 2022 Mar 31;13:1753. doi: 10.1038/s41467-022-29184-w (PMC8971480; doi:10.1038/s41467-022-29184-w)
Supplement: Supplementary file 3 — Description of Additional Supplementary Files [file 41467_2022_29184_MOESM3_ESM.pdf]

Description of Additional Supplementary Items

File name: Supplementary Video 1
Description: Explosion at NSEC on 5th September 2018 at 10:54. The explosion at 10:54:11 was recorded by several INGV network instruments (video, seismometers, infrasound arrays). The EMOT camera (thermal camera frames 1 s) video shows the Etna summit for 40 seconds (date and time at snapshot bottom). Corresponding DAS records, infrasound signal at station CARB-IF1 and seismic signal (BB, blue line) at the broadband station CAZG (Fig. 1) are also shown. The seismic velocity data is projected along the local direction of the cable and is compared with the DAS strain at channel 484 (red line).

File name: Supplementary Video 2
Description: Piano concert. Sequence of tones played with the piano, with the infrasound sensors nearby on a plate, and the desktop computer.

File name: Supplementary Video 3
Description: Small degassing events at NEC on 12 September 2018. The video shows small degassing inside the crater at ~11:00:42. The video was taken from Domenico Domanti (Guide Vulcanologiche Etna Nord). This small degassing was not recorded clearly with conventional sensors. DAS allows us to detect those events and quantify strain (Fig. 6 and 7 and Supplementary Fig. 14).
